# Absenteeism and Productivity Loss Due to Influenza or Influenza-like Illness in Adults in Europe and North America

**DOI:** 10.3390/diseases12120331

**Published:** 2024-12-17

**Authors:** David Fisman, Maarten Postma, Myron J. Levin, Joaquin Mould-Quevedo

**Affiliations:** 1 Division of Epidemiology, Dalla Lana School of Public Health, Toronto, ON M5T 3M7, Canada; david.fisman@utoronto.ca; 2 Department of Health Sciences, University Medical Center Groningen, University of Groningen, 9713 GZ Groningen, The Netherlands; m.j.postma@rug.nl; 3 Center of Excellence in Higher Education for Pharmaceutical Care Innovation, Universitas Padjadjaran, Bandung 40132, Indonesia; 4 Faculty of Economics & Business, University of Groningen, 9747 AE Groningen, The Netherlands; 5 Department of Pharmacology and Therapy, Faculty of Medicine, Universitas Airlangga, Surabaya 60115, Indonesia; 6 Departments of Pediatrics and Medicine, University of Colorado School of Medicine, Aurora, CO 80045, USA; myron.levin@cuanschutz.edu; 7 CSL Seqirus Inc., 25 Deforest Avenue, Summit, NJ 07901, USA

**Keywords:** absenteeism, caregivers, indirect cost, influenza, influenza-like illness, productivity loss

## Abstract

Healthy working-age adults are susceptible to illness or caregiving requirements resulting from annual seasonal influenza, leading to considerable societal and economic impacts. The objective of this targeted narrative review is to understand the societal burden of influenza in terms of absenteeism and productivity loss, based on the current literature. This review includes 48 studies on the impact of influenza and influenza-like illness (ILI) and reports on the effect of influenza vaccination, age, disease severity, caring for others, comorbidities, and antiviral prophylaxis on absenteeism and productivity loss due to influenza/ILI, focusing on publications originating from Canada, Europe, and the United States. Influenza/ILI results in substantial work time and productivity loss among working adults and students in Canada, Europe, and the United States, particularly those who are unvaccinated, are <65 years of age, or who have severe disease. Considerable work time and productivity loss is attributable to illness and caregiver burden related to influenza. Further research is required on the impact of influenza on absenteeism and productivity loss in adults with comorbidities to support the development of effective employer policies for working adults with underlying health conditions.

## 1. Introduction

Seasonal influenza epidemics affect a substantial proportion of the working-age population [1]. Annually, there are up to 50 million cases of influenza in Europe and 41 million cases in the United States [2,3]. Healthy adults are less susceptible to severe outcomes of influenza than older age groups, young children, and people with underlying health conditions [4,5]. However, they may still be affected by symptoms of influenza, including fever, cough, sore throat, runny nose, muscle and joint pain, and fatigue, resulting in absence from work [4,5]. Symptoms can last up to 1 week, although fatigue and cough may persist after other symptoms have resolved [4,5]. Healthy adults may also need to take time off work to care for family members with influenza infection [1]. Although people with influenza may be advised to stay at home while symptomatic to avoid transmission to others, employees may attend work while ill [6]. Presenteeism provides an opportunity to spread influenza and is associated with lost productivity from working at a reduced capacity due to the effects of illness [6,7].

Influenza virus infection is confirmed by laboratory testing. Clinical differentiation of influenza from other respiratory virus infections is difficult [4]. Thus, many studies on absenteeism and productivity loss assess influenza-like illness (ILI), either physician- or self-reported, based on the presence of a distinct set of symptoms. Although less precise than studies on confirmed influenza, data on ILI can also inform our understanding of the impact of influenza on working adults.

A recent systematic review highlighted a substantial productivity burden of influenza on the global workforce, noting that most employees still go to work with symptoms of influenza [8]. Furthermore, up to 75% of employees missed work due to influenza or ILI, and up to 75% of employed caregivers missed work to care for a household member with influenza or ILI. Therefore, the societal and economic impacts of absenteeism and productivity loss due to influenza illness and ILI are considerable [1].

The objective of this targeted narrative review is to understand the indirect burden of influenza in terms of absenteeism and productivity loss, based on the current literature, focusing on Canada, Europe, and the United States. The effects of influenza vaccination, age, disease severity, caring for others, comorbidities, and antiviral prophylaxis on absenteeism and productivity loss due to influenza/ILI were also evaluated.

## 2. Methods

### Targeted Literature Search

A targeted literature search using PubMed was performed on 11 October 2023. Search terms were as follows: influenza, productivity, presentee*, absent*, loss, lost, school, work, indirect cost, and production. English language articles published since 1 January 2000, were included. Inclusion criteria were applied to the search results in a three-stage process (stage 1: review of titles; stage 2: review of abstracts; stage 3: review of full text). Articles were included if they contained data on seasonal influenza or ILI, data from Canada, Europe, or the United States, and data on at least one pre-defined outcome of interest. Pre-defined outcomes of interest were as follows: work time lost due to influenza/ILI, productivity loss due to influenza/ILI while at work, cost of work loss due to influenza/ILI, and work loss for the care of others. The review was limited to countries in North America and Europe due to similarities in income level, vaccination programs, and healthcare systems, in order to minimize bias. Editorials and opinion articles were excluded.

Inclusion criteria were applied and data were extracted by a single reviewer. Evidence was not graded for quality. Articles meeting the inclusion criteria were grouped by pre-defined outcomes of interest, as well as by the definition of influenza or ILI used, and the population studied (general adult working populations or specific populations, such as healthcare workers [HCWs]). Data on the impact of vaccination status, age, disease severity, comorbidities, and the use of antiviral prophylaxis on these outcomes were also extracted. No specific research hypothesis was defined, and no conceptual framework was used.

## 3. Results

**Figure 1:**
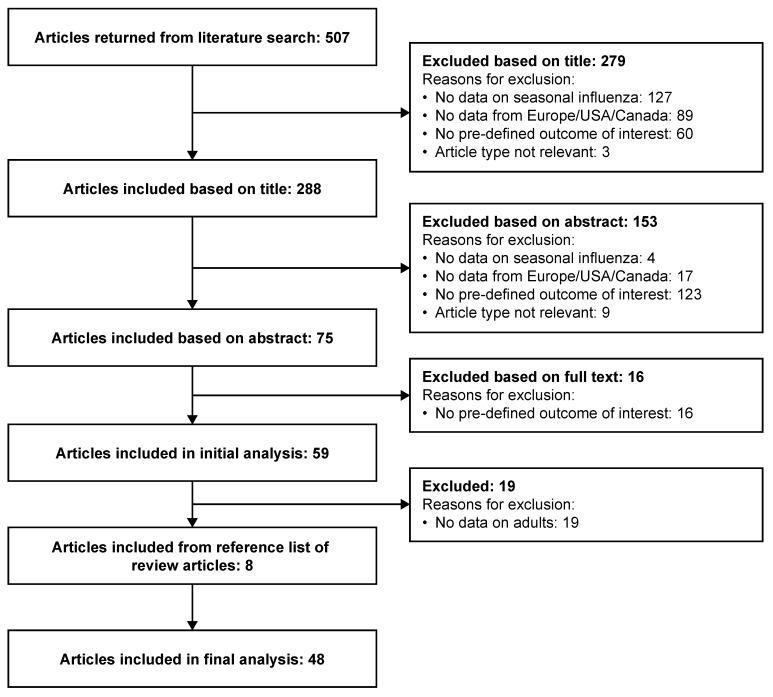
Literature search process.

The literature search returned 507 articles, of which 59 met the inclusion criteria. Of these, studies that contained data on absenteeism in school children alone (n = 19) were excluded (Figure 1). The remaining 40 articles included 39 original articles and one narrative review. A further eight articles were retrieved from the reference list of the narrative review article [1], leading to a total of 48 articles for inclusion in the final analysis.

### 3.1. Absenteeism Due to Influenza/ILI

#### 3.1.1. General Adult Populations

**Figure 2:**
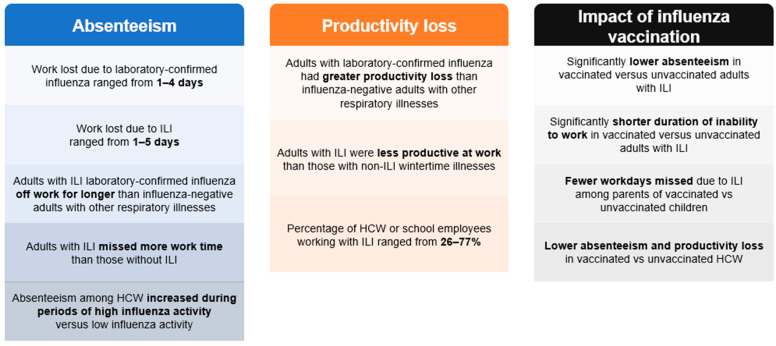
Impact of influenza and ILI on absenteeism and productivity loss among adults. Abbreviations: HCW, healthcare worker; ILI, influenza-like illness.

Studies assessing work loss due to influenza/ILI are shown in Table 1. Six studies assessed work loss due to laboratory-confirmed influenza [9,10,11,12,13,14]. Across these studies, the average days of work lost ranged from 1 to 4 (Figure 2) [11,12,13,14]. In studies that compared work time lost among people with laboratory-confirmed influenza and those who tested negative for influenza, people with confirmed influenza were off work for longer [9,11,13]. In England, among working adults (16–64 years of age), those with influenza A took more time off work or education than those who tested negative for influenza and had ILI or acute respiratory infection (ARI; 34% versus 30% and 12%). Time off work due to influenza B was close to that for influenza A and ILI (33%) [11]. In Italy, among parents of 962 children < 15 years of age, parents of children with confirmed influenza took more time off work than those with children with confirmed respiratory syncytial virus (RSV) infection (4 and 3 days of work lost by mothers and fathers of children with influenza, respectively, compared with 2 and 1 days for RSV; *p* < 0.05) [12].

Three studies used electronic health record data to assess absenteeism due to physician-diagnosed influenza [15,16,17]. Of these, two retrospective analyses of the MarketScan databases in the United States assessed absenteeism among those with influenza-related medical encounters or claims [15,16]. Across four influenza seasons, 30–37% of patients with influenza had ≥1 day of influenza-related workplace absence [16]. The mean duration of absence per person with influenza among those who took time off work ranged from 6.45 to 7.34 h across influenza seasons [16]. In an analysis of the data from the Norwegian Labour and Welfare service on sick leave classified as due to influenza according to International Classification of Primary Care (ICPC) codes, the rate of general practitioner-certified sick leave due to influenza was 1952 days per 100,000 employees (range 1595–2607) [17].

Thirteen studies assessed work loss due to ILI [11,18,19,20,21,22,23,24,25,26,27,28,29]. Definitions of ILI differed across studies. Nevertheless, individuals with ILI missed more work time than those without ILI [19,20,23,24] and those with other wintertime illnesses [11,22,24] across all studies that made these comparisons. Work time lost due to ILI ranged from 1 to 5 days (Figure 2) [11,20,21,22,24,25,26,27,29].

Three studies assessed all-cause absenteeism modeled against seasonal trends in influenza activity to estimate influenza-related absenteeism [17,30,31]. Rates of influenza-related absenteeism varied by season, ranging from 3 to 12% [17,30,31]. In a study in Canada, seasonal influenza was estimated to account for 3% of all hours lost annually [30].

#### 3.1.2. Specific Populations

Five studies assessed absenteeism due to ILI/ARI in HCWs [32,33,34,35,36]. The mean number of missed workdays was 0.47 among 1036 HCWs with ARI in a study in Canada [34]. The median number of missed workdays was 2 among 414 HCWs with ILI in a study in the United States [33] and was 4.3, on average, across 59 HCWs in a study in Switzerland [36]. Of 1180 HCWs with ILI in a second study in the United States, 71% reported an absence of >2 days [35]. All four studies that assessed all-cause absenteeism in HCWs during periods of high influenza versus low influenza activity showed that absenteeism increased during influenza seasons/epidemic periods [37,38,39,40].

One study assessed absenteeism during influenza epidemics among schoolteachers. Modeling predicted that 23–31% of the total teacher population of the Netherlands per year would be affected by influenza absenteeism [41].

### 3.2. Productivity Loss Due to Influenza/ILI

Studies assessing productivity loss with influenza/ILI (i.e., attending work while ill and working at a reduced capacity) are shown in Table 2. Two studies evaluating people with laboratory-confirmed influenza showed that individuals with influenza had greater self-assessed productivity loss than those without, or those with ARI, based on a 10-point rating scale [9,10]. In a study in France, 9% of 199 working adults with laboratory-confirmed influenza did not stop working, and the median time to return to usual work activity for those who did stop working was 7 days (95% confidence interval [CI], 7–9) [14].

Two studies evaluated productivity loss from ILI in general adult populations [21,24]. In a study in the United States, among 2013 employees, those with symptoms of ILI were significantly less productive, with a mean of 2.5 h (standard deviation [SD] 5.0) per day of reduced productivity compared with 1.1 h (SD 3.3) among those with other non-ILI wintertime illnesses (*p* < 0.001) [24]. In another study in France, median time taken to return to normal work activity among 701 people who visited a general practitioner for ILI was 7 days (range 6–8) [21].

Five studies evaluated productivity loss with ILI in specific populations (school employees or HCWs) [7,25,33,34,35]. The percentage of school employees or HCWs with ILI who worked while ill ranged from 26% to 77% (95% for ARI; Figure 2) [7,25,33,34,35].

### 3.3. Absenteeism and Productivity Loss by Subgroup

#### 3.3.1. Vaccination Status

Studies assessing work loss or productivity loss due to influenza/ILI by influenza vaccination status are shown in Table 3.

Two studies in the United States assessed work hours missed or productivity loss due to laboratory-confirmed influenza in vaccinated and unvaccinated individuals [9,10]. Neither detected any significant impact of vaccination [9,10], although a potential benefit of vaccination was observed in one study for work hours missed (20 versus 24 h; difference −4.3%; 95% CI, from −10.9 to 2.8; *p* = 0.23) and productivity loss (5.9 versus 6.1 [10-point scale]; difference −0.1; 95% CI, from −0.5 to 0.3; *p* = 0.66) [9]. In a study in Italy, parents of vaccinated children with laboratory-confirmed influenza lost significantly fewer workdays versus parents with unvaccinated children with laboratory-confirmed influenza (n = 1098; 3.22 [SD 1.86] versus 4.78 [SD 2.34] maternal work days; 0.56 [SD 0.46] versus 0.98 [SD 2.24] paternal work days; both *p* = 0.001) [13]. In a study in the United States, more patients with influenza-related medical encounters in the MarketScan database had at least 1 day of work absence during vaccine-mismatched seasons versus matched seasons (26.0% versus 33.3%) [16].

Among studies assessing work loss due to ILI or all-cause absenteeism during influenza epidemics, several studies detected statistically significant differences between vaccinated and unvaccinated individuals [42,43,44]. However, in one study of 3663 workers in France, no statistically significant difference in the average number of days of sick leave among those absent for any medical cause was observed between vaccinated and unvaccinated employees [28]. In a Swiss study that assessed productivity loss in workers with ILI, vaccinated people had significantly shorter durations of inability to work than unvaccinated people (2.79 days [95% CI, 2.51–3.08] versus 3.22 days [95% CI, 3.16–3.28], *p* = 0.008) [27].

Two studies showed significant reductions in workdays missed due to ILI among household contacts of children from schools offering influenza vaccination versus those attending either of two control schools where vaccination was not offered (study 1: 1.3 versus 7.0 [school 1] and 5.7 [school 2] workdays lost, *p* = 0.0004; study 2: mean 0.292 versus 0.388 days, difference 0.07 [95% CI, 0–0.14], *p* = 0.04 [includes time taken off work to care for sick children]) [45,46]. Similarly, in another study of 23,014 working adults with household children, the prevalence of workdays lost in households with a vaccinated child was 0.92 times (95% CI, 0.80–1.06) that of households with an unvaccinated child. This difference was statistically significant when using a model that included paid sick leave (0.79 times; 95% CI, 0.67–0.93) [47].

Studies of HCWs noted potentially lower absenteeism rates and productivity loss in vaccinated versus unvaccinated workers [33,36,48]. Among a convenience sample of 341 HCWs, 3.4% of vaccinated HCWs were absent for more than 1 day due to ILI, compared with 13% of unvaccinated HCWs (*p* ≤ 0.006) [48]. Among a randomized sample of 200 university hospital employees, absenteeism rates due to ILI were 17% in vaccinated HCWs and 25% in unvaccinated HCWs (odds ratio 0.6; 95% CI, 0.2–1.7) [36]. In a survey of 1914 HCWs, 414 reported an ILI and 183 reported working with an ILI. Of these, 44.6% who reported working with an ILI had received an influenza vaccination, compared with 29.3% who were not vaccinated (*p* = 0.03) [33].

Several studies assessed all-cause absenteeism in vaccinated versus unvaccinated HCWs during influenza epidemic periods [37,38,39,40,49,50]. Significantly greater absenteeism rates were observed in unvaccinated HCWs across all studies [37,38,39,40,49,50].

**Table 3:** Studies on absenteeism or productivity loss by vaccine status.

Publication	Location	Time Period	Study Design and Participants	Definition of Illness	Results
**General adult populations: laboratory-confirmed influenza**					
Petrie et al., 2016 [9]	United States	2012–2013	Prospective study of working adults (N = 1548)	Laboratory-confirmed influenza versus influenza negative	No significant modifications of work hours missed or work productivity loss were noted for vaccinated individuals versus non-vaccinated individuals
Van Wormer et al., 2017 [10]	United States	2012–2016	Analysis of data from participants in vaccine effectiveness studies (N = 1278)	PCR-confirmed influenza-positive versus confirmed influenza-negative people with ARI	No significant productivity difference between those with versus without seasonal influenza vaccination for both influenza-positive and influenza-negative participants
Principi et al., 2003 [13]	Italy	2001–2002	Study of children presenting to emergency department (N = 3771) and their household contacts	Laboratory-confirmed influenza	Mothers of vaccinated children had fewer workdays lost than mothers of unvaccinated children (mean 3.22 [SD 1.86] versus 4.78 [SD 2.34], VE 33%, *p* = 0.001) Fathers of vaccinated children had fewer workdays lost than fathers of unvaccinated children (mean 0.56 [SD 0.46] versus 0.98 [SD 2.24], VE 43%, *p* = 0.001)
**General adult populations: physician-diagnosed influenza/ILI**					
Karve et al., 2013 [16]	United States	2000–2009	Retrospective analysis of employee data from MarketScan databases	Influenza-related medical encounters with temporal co-occurrence of workplace absence	A larger proportion of patients had at least 1 day of influenza B-related workplace absence during vaccine-mismatched seasons than patients in matched seasons (26.0% versus 33.3%) Higher workplace absence hours were observed during mismatched seasons than during matched seasons (mean: 6.8 h versus 5.1 h)
**General adult populations: ILI**					
Nichol et al., 2008 [42]	United States	2002–2003	Survey of university students (N = 19,796)	≥1 upper respiratory tract infection symptom (feverishness, chills, muscle aches, headache, sore throat, cough, runny nose or temperature of >38 °C)	Mean number of days of work missed was 0.21 (SD 0.96) in vaccinated participants and 0.29 (SD 1.08) in unvaccinated participants (adjusted difference −0.09, 95% CI, from −0.13 to −0.05) Number needed to vaccinate to prevent 1 day of missed work was 11
Bridges et al., 2000 [43]	United States	1997–1999	Double-blind, placebo-controlled trial of inactivated influenza vaccine (N = 1184)	ILI, defined as feverishness or temperature of 37.7 °C plus cough or sore throat (CDC definition)	During the 1997/1998 season, vaccine recipients reported significantly more lost workdays than placebo recipients (rate 0.290 versus 0.200, difference −45%, *p* = 0.047) In total, 45 vaccine recipients (rate 0.078) and 51 placebo recipients (rate 0.092) had any lost workdays (difference 15%, *p* = 0.15) During the 1998/1999 season, vaccine recipients reported fewer lost workdays than placebo recipients (rate 0.082 versus 0.121, difference 32%, *p* = 0.002) Fewer vaccine recipients (n = 45, rate 0.077) had any lost workdays versus placebo recipients (n = 69, rate 0.116; difference 34%, *p* < 0.001)
Millot et al., 2002 [28]	France	1996–1997	Survey of employees (N = 3663)	ILI, defined as association of fever with sudden systemic symptom and ≥1 respiratory sign	Vaccinated individuals were not absent from work more often than unvaccinated individuals (90/301 [29.9%] versus 1096/3362 [32.6%]) Average number of days sick leave was not significantly different between vaccinated and unvaccinated workers (2313/301, 7.7 [SD 27.61] versus 18,315/3362, 5.4 [SD 19.05], t = 1.9, *p* = 0.06) when all medical causes were taken together
King et al., 2005 [45]	United States	2003	Controlled intervention study of schools with/without offer of influenza vaccination	Fever or respiratory illness	Households with children attending a school where live attenuated influenza vaccine was offered had significant relative reductions in days of paid work missed compared with control households with children attending two schools where vaccination was not offered (mean rate of paid workdays lost 1.3 days versus 7.0 and 5.7 days per 100 adults, *p* = 0.0004)
King et al., 2006 [46]	United States	2004–2005	Controlled intervention study of schools with/without offer of influenza vaccination	ILI, defined as fever or respiratory illness that included runny nose, nasal congestion, sinus problems, earache, ear infection, cough, sore throat, muscle aches, chills, or wheezing	Households with children in schools offering influenza vaccination reported significantly fewer workdays missed by parents to care for their own or someone else’s ILI, when compared with households with children in non-intervention schools (0.292 versus 0.388, difference 0.07 [95% CI, 0–0.14], *p* = 0.04)
Tomonaga et al., 2021 [27]	Switzerland	2016–2017	Absenteeism data from Swiss Sentinel Surveillance Network of the Swiss Federal Office of Public Health (SFOPH)	ILI, defined as sudden onset of high fever >38 °C and cough or sore throat, as well as secondary illnesses after influenza	In a subgroup of patients in Sentinel reports, vaccinated individuals had a significantly shorter duration of inability to work compared with all unvaccinated individuals (2.79 days [95% CI, 2.51–3.08] versus 3.22 days [95% CI, 3.16–3.28]; *p* = 0.008) The estimated total costs related to productivity loss would decrease by CHF 18 million (−15.3%) in 2016 and by CHF 15 million in 2017 (−14.2%) if all persons were vaccinated
**General adult populations: all-cause absenteeism and influenza/ILI surveillance trends**					
Bleser et al., 2019 [47]	United States	2013–2015	National Health Interview Surveys from working adults with children (N = 24,014)	All sick days (households with vaccinated versus unvaccinated children)	Having a vaccinated child in the household was significantly associated with fewer workdays lost in one of three models: — Overall: 0.92 (95% CI, 0.80–1.06) times the prevalence of work loss days versus having no vaccinated child — Model with no paid sick leave: 1.10 (95% CI, 0.87–1.38) — Model with paid sick leave: 0.79 (95% CI, 0.67–0.93)
Ferro et al., 2020 [44]	Italy	2017–2018	Observational cohort study of employees of a manufacturing company (N = 408)	Comparison of absenteeism during influenza epidemic and non-epidemic periods	During the influenza period, the monthly mean sick leave days per employee was significantly lower among vaccinated versus unvaccinated workers (0.328 days/person versus 0.752 days/person, *p* = 0.022)
**Specific populations: ILI**					
Chiu et al., 2017 [33]	United States	2014–2015	Survey of HCWs (N = 1914)	ILI, defined as fever, sore throat, or cough	Of HCWs who reported working with ILI, 44.6% were vaccinated and 29.2% were unvaccinated (*p* = 0.03)
Speroni et al., 2005 [48]	United States	2004–2005	Convenience sample of HCWs (N = 341)	Specific symptoms (fever, headache, extreme tiredness, dry cough, sore throat, runny nose, stuffy nose, muscle aches)	>1 day of absenteeism as a result of >1 influenza symptom was reported by 3.4% of vaccinated individuals and 13% of unvaccinated individuals (chi-square 10.4, *p* ≤ 0.006) However, the average number of days absent was highest among individuals who received FluMist (4.5 days) versus Fluzone (1.9 days) and unvaccinated individuals (2.1 days)
Szucs et al., 2001 [36]	Switzerland	1999–2000	HCW survey (N = 200)	ILI	Absenteeism rates were 17% in vaccinated individuals and 25% in unvaccinated individuals (odds ratio 0.6; 95% CI, 0.2–1.7)
**Specific populations: all-cause absenteeism and influenza/ILI surveillance trends**					
Van Buynder et al., 2015 [37]	Canada	2012–2013	Retrospective cohort study of HCWs	All-cause sick hours	Mean rates of absenteeism for unvaccinated and vaccinated workers pre-influenza season were 5.16 and 4.45 days; mean rates during the influenza season were 6.26 and 5.01 days The rates of sick hours were significantly different between those vaccinated and unvaccinated during both time periods (*p* < 0.000) Absenteeism increased by 1.10 h per 100 scheduled work hours from pre- to during the influenza season for unvaccinated workers, and by 0.56 for vaccinated workers Linear regression showed that unvaccinated HCWs had an increased rate of absenteeism of 0.5 (95% CI, 0.2–0.9) hours/per 100 scheduled hours, compared with those vaccinated (*p* = 0.004)
Gianino et al., 2021 [40]	Italy	2010–2013 2017–2018	HCWs absenteeism data from hospital database	Excess absenteeism in severe influenza season compared with non-epidemic period and with three moderate influenza seasons	The rate of days of work lost was almost half for vaccinated versus non-vaccinated personnel during the peak of the influenza epidemic (0.22 versus 0.40, *p* = 0.02) Vaccinated workers had lower excess absenteeism in comparison with non-vaccinated workers (1.74 versus 2.71 days/person) when compared against their baseline (non-epidemic period)
Zaffina et al., 2019 [39]	Italy	2016–2018	Retrospective observational study of HCWs (N = 2090–2097 across time periods studied)	Comparison of absenteeism during influenza epidemic and non-epidemic periods	Absenteeism rate increased by 0.38 (2.00 [95% CI, 1.86–2.14] versus 1.62 [95% CI, 1.35–1.89], *p* = 0.03) during the 2016–2017 epidemic period among unvaccinated compared with vaccinated workers Absenteeism rate increased by 0.46 (2.06 [95% CI, 1.86–2.14] versus 1.60 [95% CI, 1.86–2.14], *p* = 0.01) during the 2017–2018 epidemic period among unvaccinated compared with vaccinated workers
Antinolfi et al., 2020 [49]	Italy	2017–2018	Retrospective study of HCWs (N = 4382)	Comparison of absenteeism during influenza epidemic and non-epidemic periods	During the influenza season, non-vaccinated HCWs lost 2.47 person-days/100 person-days of work, compared with 1.92 person-days/100 person-days of work among vaccinated HCWs (*p* < 0.001)
Gianino et al., 2017 [38]	Italy	2010–2013	Hospital database analysis (N = 5291–5544 across years studied)	Comparison of absenteeism during influenza epidemic and non-epidemic periods	The absenteeism rate among workers without vaccination during the epidemic periods was approximately 1.5 times higher than the rate observed among vaccinated employees (absolute and relative increases 2.01 days/person and approximately 70%, respectively)
Costantino et al., 2020 [51]	Italy	2007–2019	Hospital data on absenteeism among HCWs	Acute sickness during influenza seasons, pre-, and post-interventions to increase vaccine coverage	Average number absent from work was 1858 (95% CI, 1797–1919) during pre-intervention seasons and 1693 (95% CI, 1573–1813) during post-intervention seasons (8.8% reduction) Average number of working days lost was 11,571 (95% CI, 11,023–12,119) during pre-intervention seasons and 10,077 (95% CI, 8626–11,528) during post-intervention seasons (12.9% reduction) Average number of working days lost per worker was 4.5 (95% CI, 4.3–4.7) during pre-intervention and 4.0 (95% CI, 3.4–4.6) during post-intervention seasons (11.1% reduction)
Murti et al., 2019 [50]	Canada	2012–2017	HCW payroll/absenteeism data (N = 107,258)	All sick time	Workers who reported ‘early’ (OR 0.874, 95% CI, 0.866–0.881) and ‘late’ (OR 0.969, 95% CI, 0.954–0.985) influenza vaccination were both significantly less likely to have any sick time during influenza seasons compared with workers who did not report vaccination Workers who reported ‘early’ vaccination had a significantly lower monthly sick rate during influenza seasons (RR 0.907; 95% CI, 0.901–0.912) compared with those who did not report vaccination; however, there was no significant difference for those who reported ‘late’ vaccination (RR 0.966; 95% CI, 0.986–1.007) Overall, sick rates were similar among HCWs, regardless of whether they changed from ‘early’ vaccination in one year to ‘not reported’ in another year, or vice versa
Huiberts et al., 2022 [41]	The Netherlands	2016–2019	Data on registered schoolteacher sick leave, vaccine effectiveness. and incidence of ILI in primary care	Self-reported influenza sick leave	Nationally, a vaccine uptake of 2% would prevent 128–350 absenteeism notifications and 447–1156 absent days A vaccine uptake of 50% would prevent 3195–8756 absenteeism notifications and 11,178–28,896 absent days The number needed to vaccinate to prevent one influenza absenteeism notification ranged from 11.6 to 31.9 The number needed to vaccinate to prevent 1 working day lost to influenza absenteeism ranged from 3.5 to 9.1

Abbreviations: ARI, acute respiratory infection; CDC, US Centers for Disease Control and Prevention; CI, confidence interval; HCW, healthcare worker; ILI, influenza-like illness; OR, odds ratio; PCR, polymerase chain reaction; RR, risk ratio; SD, standard deviation; SFOPH, Swiss Federal Office of Public Health; VE, vaccine effectiveness.

#### 3.3.2. Age Group

Nine studies evaluated work loss due to influenza/ILI by age group [10,11,16,27,30,33,34,52,53]. Generally, in studies of general adult populations, less work time was lost, and productivity was reduced to a lesser extent, in older versus younger adults. In a survey of working Canadian adults with diagnosed influenza or self-reported ILI, those who were 50–64 years of age were more likely than those ≥65 years of age to be absent from work (*p* = 0.009 in 2019/2020) or to be present but working at low capacity (*p* = 0.02 in 2018/2019) [52]. In a community cohort study of 2919 adults in England, a smaller percentage of adults ≥65 years of age than 16–64 years of age took time off for work/education due to ILI (9% versus 26%), influenza A (13% versus 31%), and influenza B (0% versus 20%); however, no statistical comparisons were made [11]. In Switzerland, among 3971 cases of ILI, the mean number of workdays lost per case was similar across age classes <65 years of age and was lower than average in those ≥65 years of age [27]. Younger age groups had a higher rate of cases of inability to work than older age groups. In a retrospective analysis of the MarketScan databases, individuals 45–64 years of age had higher influenza-related workplace absence hours than those 25–44 years of age (range 7.6–9.7 versus 5.2–6.6). Similarly, individuals 45–64 years of age had the highest influenza-related productivity loss costs, followed by those in the 25–44 years of age group, for all four influenza seasons studied [16]. A study from the Canadian Labour Force Survey found that the level of influenza activity was associated with hours lost in younger age groups (18–44 years of age) only, while the proportion of hours lost due to any illness or disability increased significantly with age [30].

Among HCWs, work loss and productivity work loss generally did not differ by age. In an online survey of 220 employees/trainees at a medical center in the United States, the percentage of workers who reported presenting to work with ILI was not significantly associated with age group [7]. Among 2093 HCWs in Canada, a multivariable analysis showed no difference in the number working with ARI across age groups [34]. Among 1914 HCWs in the United States, the percentage who reported working with ILI did not significantly differ by age group (18–34 years, 42.3%; 35–49 years, 45.6% [*p* = 0.68 versus 18–34 years]; ≥50 years, 34.7% [*p* = 0.33 versus 18–34 years]) [33]. In a study involving 5401 HCWs in Italy, there was a significant increase in sick leave during seasonal influenza epidemics for all age ranges [53].

#### 3.3.3. Disease Severity

Four studies evaluated the impact of disease severity on work time lost or productivity loss due to influenza/ILI; all suggested a link between increased severity and more work time lost [14,27,34,40]. In a study of the Swiss workforce, patients reporting a high fever (>38 °C) had a significantly longer duration of inability to work (3.37 days [95% CI, 3.19–3.56] versus 3.05 days [95% CI, 2.79–3.30] for people without a high fever; *p* = 0.014) [27]. In a survey of household contacts in France, the number of lost workdays was correlated with the symptom score on the first day of illness (r = 0.45, *p* < 0.001) [14]. Among 5287 HCWs in Italy, a significantly greater excess of absenteeism was observed during a severe influenza season compared with a moderate influenza season (+0.75 days/person, *p* = 0.03) [40]. One study in 2093 Canadian HCWs demonstrated that productivity loss with ARI increased with increasing symptom severity score [34].

#### 3.3.4. Caring for Others

Caring for others with influenza/ILI contributed to work time loss. Among households with medical insurance coverage and school-age children in the United States, households with ILI missed 0.89 (95% CI, 0.48–1.30) more workdays while caring for ill household members versus households without ILI [23]. In a survey of 161 parents or guardians in England, mean work absence to care for children with ILI (n = 34) was 3.7 days (95% CI, 2.7–4.8) [26]. In a survey of school children in the United States, for every 100 children, influenza accounted for an estimated 20 days of work missed by parents [19]. Another survey of 954 parents of school children reported that an adult missed work to care for an ill child in 53% of families [18].

Across studies, the percentage of cases of work loss attributed to caring for others ranged from 1% to 9% [11,17,27]. In the community cohort study of 2919 adults in England, the percentage of illnesses during which a working adult took time off from work or education to care for an ill working adult was 13% for influenza A and 0% for influenza B [11]. In a study of the Swiss workforce, 90% of cases of inability to work due to influenza/ILI were due to a person’s own illness, 1–2% of cases were due to caregiving, and 8–9% of cases were a combination of both [27]. In Norway, approximately 4–5% of work absences were attributed to care for sick children with influenza [17].

#### 3.3.5. Comorbidities

Data on the impact of comorbidities on work loss due to influenza/ILI are limited. In one retrospective study of the MarketScan databases, among influenza cases with comorbid asthma, more than 36% had at least 1 day of influenza-related workplace absence for each of the four influenza seasons [16]. In a survey of 412 employees in the United States, those with a weakened immune system caused by cancer, chronic illness, or medications were less likely to report working while ill than those without (20.0% versus 78.7%; *p* = 0.01); diabetes and asthma were not associated with working while ill [25]. In employees in Switzerland, increased risk of complications and medication had no significant impact on the duration of their inability to work [27].

#### 3.3.6. Prophylaxis

One study assessed the impact of prophylaxis on work time lost due to influenza/ILI [54]. In this study of 800 HCWs in the United States, offering oseltamivir prophylaxis to exposed HCWs had no apparent impact on overall absenteeism rates or duration of sick leave compared with the previous influenza season, during which only exposed, unvaccinated HCWs were offered prophylaxis [54].

### 3.4. Cost of Work Loss Due to Influenza/ILI

Studies evaluating the cost of work time lost due to influenza/ILI are shown in Table 4. In the United States, the cost of productivity loss was higher among people with influenza-related medical encounters in the MarketScan database during vaccine-mismatched seasons compared with matched seasons (USD 51,483 versus USD 31,454 per 100,000 members) [15]. Consistent with this, three studies noted higher costs of absenteeism among unvaccinated compared with vaccinated employees or HCWs [39,43,44]. Other factors associated with increased cost due to work time lost included younger age [27,53], the presence of comorbidities [16], and caring for family members with ILI [23].

## 4. Discussion

This targeted review suggests an association between influenza/ILI and work/productivity loss. Work time lost and productivity loss due to laboratory-confirmed influenza were consistently higher than work loss because of other respiratory diseases and wintertime illnesses compared to people without influenza [9,10,11,12,13,27]. The majority of studies assessed work time lost due to ILI and demonstrated that individuals with ILI missed more work time and were less productive than those without ILI or with other wintertime illnesses [20,22,23,24]. Among specific populations (HCWs and school employees), ILI was associated with considerable work time and productivity loss [25,32,33,34,35], and absenteeism was higher during periods of high influenza versus low influenza activity [37,38,39,40]. These findings suggest that ILI is an appropriate surrogate for laboratory-confirmed influenza when assessing absenteeism and productivity loss.

Most studies that assessed the impact of vaccination on absenteeism demonstrated less work time and productivity loss, as well as lower costs, among vaccinated versus unvaccinated individuals [9,13,27,42,43,44], members of households with vaccinated children versus those with unvaccinated children [45,46,47], or during vaccine-mismatched seasons [16]. As influenza vaccination has been shown to reduce the rate of infection and severity of disease [4], the impact of vaccination on absenteeism and productivity loss is not unexpected. Several studies document that greater disease severity was associated with increased work time lost or productivity loss [14,27,34,40].

Work time and productivity losses were generally higher in adults younger than 60–65 years of age than in older adults [10,11,27,52]. This may have been due to a smaller proportion of older adults being in work; however, the same trend was observed in a study that included working adults only [52]. Younger age groups are likely to have higher exposure to influenza, resulting from the presence of children in the household acting as a natural reservoir, as well as greater time spent working or out of the household at events and gatherings than older adults. Caring for others with influenza/ILI also contributed to work time lost by the caregiver [11,17,18,19,23,26,27], which may disproportionately affect younger adults. Furthermore, older adults may have been more likely to have been vaccinated against influenza, as, in many countries, vaccination is only recommended for adults >65 years of age and for those with risk conditions or high exposure to infection [4].

Data on the impact of comorbidities on work time and productivity loss due to influenza/ILI were limited. People with certain underlying health conditions, including asthma, chronic lung, heart, or kidney disease, diabetes, and immunocompromising conditions or medications are known to be at high risk of developing serious complications of influenza infection [4,55]. Further research is warranted to determine whether specific comorbidities may be particularly associated with increased absenteeism or productivity loss.

The findings of this review are generally consistent with other reviews that have included articles from outside of Canada, Europe, and the United States. Overall, the average days of work lost across studies assessing laboratory-confirmed influenza ranged from 1 to 4 [11,12,13,14]. This is comparable to values seen worldwide in the narrative literature review by Keech et al., which estimated 1.5–4.9 working days lost due to laboratory-confirmed influenza [1]. Similarly, in a recent systematic literature review, mean work time lost due to influenza/ILI ranged from <1 to >10 days, but was often reported to be approximately 2–3 days [8].

Limitations of this review include the small number of articles on each pre-defined outcome of interest. Certain countries were overrepresented (e.g., the United States, Italy, France, Switzerland, and Canada), while no relevant studies from Spain or Germany were identified. Overall, the literature search identified only 48 articles for inclusion, highlighting a need for further research on this topic, No meta-analyses were performed, and no quality assessment was carried out. As the majority of the studies included were small observational studies, the quality of the methodology may have been low, representing a risk of bias. In addition, while influenza was consistently defined, the definitions of ILI and ARI differed by study in terms of the symptoms included, and some did not state the definition used. As differences were minimal and the countries included were similar in terms of income level and vaccination programs, combining conclusions from these studies was warranted. However, care should be taken when making direct comparisons across studies, given the differences in study designs.

## 5. Conclusions

Influenza/ILI has been shown to result in significant work time and productivity losses among working adults in Canada, Europe, and the United States, particularly those who are unvaccinated, are <65 or <60 years of age, or who have severe disease. Further research is required on the impact of influenza on absenteeism and productivity loss in adults with comorbidities to support the development of effective employer policies for working adults with underlying health conditions. Research into the impact of vaccination on absenteeism and productivity loss could support the economic and health benefits of vaccination and facilitate our understanding of the potential benefits of extending vaccination programs to wider adult populations.

## Figures and Tables

**Table 1:** Studies on work loss due to influenza/ILI.

Publication	Location	Time Period	Study Design and Participants	Definition of Illness	Results
**General adult populations: laboratory-confirmed influenza**					
Petrie et al., 2016 [9]	United States	2012–2013	Prospective study of working adults (N = 1548)	Laboratory-confirmed influenza versus influenza-negative	Follow-up survey showed that influenza cases missed 45% more work hours (20.5 h [range 12–32] versus 15.0 h [range 4–24]; *p* < 0.001) than non-cases based on a subjective measurement scale (range 0–10)
Van Wormer et al., 2017 [10]	United States	2012–2016	Analysis of data from participants in vaccine effectiveness studies (N = 1278)	PCR-confirmed influenza-positive versus confirmed influenza-negative people with ARI	Influenza-positive adults lost 69% of work hours between illness onset and follow-up, and influenza-negative adults lost 58% of work hours between illness onset and follow-up
Fragaszy et al., 2018 [11]	England	2006–2011	Community cohort study (N = 2919)	PCR-confirmed influenza	Percentage of ill working adults (≥16 years of age) taking time off work or education was 34% for influenza A and 33% for influenza B Average number of days taken off work was 4.0 (1–9) and 3.0 (2–4), respectively Percentage of illnesses whereby someone took time off work or education to care for an ill working adult (≥16 years of age) was 13% for influenza A and 0% for influenza B Average number of days taken off work to care for an ill working adult was 2.3 (2–3) and 0, respectively
Esposito et al., 2005 [12]	Italy	2002–2003	Prospective study of children attending emergency department (N = 1520) and their families	Laboratory-confirmed influenza	Mothers of children with influenza missed a median of 4 (range 1–9) days of work, versus 2 (range 2–5) days for mothers of children with RSV (*p* < 0.05) Fathers of children with influenza missed a median of 3 (range 2–8) days of work versus 1 (range 1–4) for fathers of children with RSV (*p* < 0.05)
Principi et al., 2003 [13]	Italy	2001–2002	Study of children presenting to emergency department (N = 3771) and their household contacts	Laboratory-confirmed influenza	There were significantly more missed workdays among parents of those with laboratory-confirmed influenza (n = 915, mean 1.39 ± SD 3.09) versus those without (n = 9128, mean 0.59 ± SD 2.02)
Carrat et al., 2002 [14]	France	2000	Household contact study	Laboratory-confirmed influenza	Among 199 index cases with influenza, mean lost workdays were 4.0 (SD 2.8)
**General adult populations: physician-diagnosed**					
Karve et al., 2013 [15]	United States	2000–2009	Retrospective analysis of employee data from MarketScan databases	Influenza-related medical encounters with temporal co-occurrence of workplace absence	The average per-person influenza B-related workplace absence was 6.0 h
Karve et al., 2013 [16]	United States	2005–2009	Retrospective analysis of employee data from MarketScan databases	≥1 medical claim with diagnosis of influenza	In total, 30–37% of patients had ≥1 days of influenza-related workplace absence Patients had approximately 7 h of workplace absence due to influenza (range across influenza seasons: 6.5–7.3 h)
De Blasio et al., 2012 [17]	Norway	2005–2010	National registry data on GP-certified sick leave	ICPC-2 diagnosis of influenza (R80)	GP-certified sick leave rate due to influenza based on registry data was 1952 (range across influenza seasons 1595–2607) days per 100,000 employees
**General adult populations: ILI**					
Nettleman et al., 2001 [18]	United States	1997–1998	Survey of parents of schoolchildren (N = 954)	Respiratory illness, excluding asthma	An adult missed work to care for an ill child in 53% of families
Neuzil et al., 2002 [19]	United States	2000–2001 influenza season	Survey of parents of school children (N = 216)	Fever, coryza, sore throat, cough, myalgia, earache, headache, nausea, vomiting, diarrhea	Compared with a non-influenza season, for every 100 children, influenza accounted for 19.8 excess days of work missed by parents
Akazawa et al., 2003 [20]	United States	January–July 1996	Medical Expenditure Panel Survey Household component data (N = 7037)	ILI	The mean number of health-related missed workdays was 1.81 (SD 7.36, range 0–180) Regression analysis suggested individuals with ILI missed 1.30 days more work than those without ILI
Carrat et al., 2004 [21]	France	1999–2000 influenza season	Observational household contact study (N = 701)	ILI	Mean number of workdays lost was 3.7 (SD 2.7) if antibiotics were taken and 3.8 (SD 2.8) if antibiotics were not taken
Nichol et al., 2005 [22]	United States	October 2002–April 2003	Cohort study of college students (N = 4919)	ILI	Students with ILIs missed ~3 times as many days of work compared with students with colds (range 0.77–1.10 days across each month versus 0.24–0.34 days across each month of the influenza season)
Li et al., 2007 [23]	United States	1996–2002	Medical Expenditure Panel Survey (N = 12,850 households)	ILI	Employed members of households reporting ILI lost 1.12 (95% CI, 0.20–2.04) more workdays versus those without ILI
Palmer et al., 2010 [24]	United States	2007–2008 influenza season	Observational cohort study of employees (N = 2013)	ILI (modified CDC definition)	A significantly greater proportion of employees with ILI had ≥1 days of absence versus those with no ARI symptoms (72% versus 30%, *p* < 0.001) Employees with a household member with ILI missed more days of work than those with household members with other wintertime illnesses (0.9 versus 0.3 days, *p* < 0.001) On average, employees with at least one household member who had an ILI during the study period attributed 0.5 missed days of work specifically to a household member with an ARI Employee ILI accounted for 15% of all absenteeism, and household ILI accounted for 6%
De Perio et al., 2014 [25]	United States	2013	Survey of school employees (N = 412)	ILI, defined as being sick with fever and either sore throat or cough	Median time taken off work because of ILI was 1 day (range 0–7 days)
Thorrington et al., 2017 [26]	England	2012–2014	Survey of parents/guardians (N = 161)	ILI, ECDC definition	Caregivers of sick children were absent from work for a total time of 3.7 days (95% CI, 2.7–4.8)
Fragaszy et al., 2018 [11]	England	2006–2011	Community cohort study (N = 2919)	ILI, defined as fever ≥ 37.8 °C or feverish symptoms and either cough or sore throat, PCR-confirmed influenza	The percentage of ill working adults (≥16 years of age) taking time off for work or education was 12% for ARI and 30% for ILI The average number of days taken off was 2.6 (range 1–14) and 3.3 (1–18), respectively The percentage of illnesses in which someone took time off from work or education to care for an ill working adult was 4% for ARI and 6% for ILI The average number of days taken off to care for an ill working adult was 1.2 (range 1–2) and 1.2 (1–3), respectively
Tomonaga et al., 2021 [27]	Switzerland	2016–2017	Absenteeism data from Swiss Sentinel Surveillance Network of the Swiss Federal Office of Public Health (SFOPH)	ILI, defined as sudden onset of high fever >38 °C and cough or sore throat, as well as secondary illnesses after influenza	Total yearly cases of inability to work due to ILI was 101,287 in 2016 and 86,373 in 2017 Mean duration of absence was 3.20 days (95% CI, 3.12–3.27) in 2016 and 3.22 days (95% CI, 3.14–3.31) in 2017 Total number of workdays lost was 0.04576 per inhabitant in 2016 and 0.03899 per inhabitant in 2017 Persons with a positive influenza test result (n = 182, 4.6%) had a significantly longer duration of inability to work compared with those with a negative influenza test: 3.32 days (95% CI, 3.12–3.54) versus 3.08 days (95% CI, 2.90–3.27), *p* = 0.039
Millot et al., 2002 [28]	France	1996–1997	Survey of employees (N = 3663)	ILI, defined as association of fever with sudden systemic symptom and ≥1 respiratory sign (clinical or self-diagnosis)	In total, 1186 (32.4%) workers with ILI took sick leave during the 7-month period (total 20,628 days)
Sessa et al., 2001 [29]	Italy	1998–1999	Cases of GP-diagnosed influenza followed up for outcome data (N = 6057)	Clinical influenza (GP diagnosis)	Employees lost a mean of 4.9 (SD 1.6) and median of 5 (range 1–8) days of work Self-employed workers lost a mean of 4.3 (SD 1.7) and median of 4 (range 1–8) days of work
**General adult populations: all-cause absenteeism and influenza/ILI surveillance trends**					
De Blasio et al., 2012 [17]	Norway	2005–2010	Work absence data from Norwegian postal service	All-cause absenteeism (influenza attributable work loss estimated using Poisson regression based on ILI rates in the population)	Mean self-certified work loss due to influenza at Norway Post was 4363 (95% CI, 2500–6238) days annually, corresponding to 10.3% of total absences Mean self-certified absence rate due to influenza was 132 (95% CI, 75–188) per 100,000 working days Mean GP-certified work loss due to influenza at Norway Post was 7295 (95% CI, 1702–17,204) days annually, corresponding to 2.7% of total absences Mean GP-certified absence rate due to influenza was 219 absences (95% CI, 51–514) per 100,000 working days Seasonal influenza accounted for approximately 4–5% of work absence related to care for sick children
Schanzer et al., 2011 [30]	Canada	1995–2009	Time series using data from Statistics Canada’s Labour Force Survey	All-cause absenteeism (influenza-attributable absenteeism modeled based on percentage of tests positive for influenza during each season and weekly/monthly trends in influenza activity)	Absenteeism rates for seasonal influenza averaged 12% from the 1997/1998 to 2008/2009 seasons An average of 0.08% (95% CI, 0.06–0.10) of hours worked were lost annually due to seasonal influenza Seasonal influenza accounted for 3% of all hours lost annually Rates of absenteeism varied by season, with higher rates associated with seasons during which more than one distinct antigenic strain circulated
Groenewold et al., 2019 [31]	United States	2017–2018	Workplace absenteeism surveillance (CDC Current Population Survey)	All-cause absenteeism among full-time workers during influenza epidemic	Prevalence of absenteeism peaked at 3.0% (95% CI, 2.8–3.2), which significantly exceeded the epidemic threshold
**Specific populations: ILI**					
Sartor et al., 2002 [32]	France	1999	Healthcare workers present during in-hospital influenza outbreak (N = 22)	ILI	The outbreak resulted in staff members taking 14 person-days of sick leave
Chiu et al., 2017 [33]	United States	2014–2015	Survey of healthcare workers (N = 1914)	ILI, defined as fever, sore throat, or cough	The median number of missed workdays among workers with ILI was 2 days (range 0–30; n = 414)
Jiang et al., 2019 [34]	Canada	2010–2014	Active surveillance of healthcare workers (N = 2093)	ARI (shortness of breath, cough, sore or scratchy throat, or coryza)	Mean 0.47 (95% CI, 0.45–0.49) days of absence per ARI per participant with ARI symptoms (n = 1036)
Hoang Johnson et al., 2021 [35]	United States	2017–2018	Healthcare worker survey (N = 2391)	ILI, defined as symptoms of fever, chills, cough, or sore throat	Of workers who stayed at home with ILI (n = 1180), 71% reported an absence of >2 days
Szucs et al., 2001 [36]	Switzerland	1999–2000	Healthcare worker survey (N = 200)	ILI	On average, employees were off work for 4.3 (95% CI, 3.5–5.1) days (n = 59) with ILI Total lost working days for the hospital estimated at 3096–9079 per season for ILI and 646–1943 for influenza infection
**Specific populations: all-cause absenteeism and influenza/ILI surveillance trends**					
Van Buynder et al., 2015 [37]	Canada	2012–2013	Retrospective cohort study of healthcare workers	All-cause absenteeism pre- and during influenza seasons	Absenteeism increased by 0.69 h per 100 scheduled work hours from pre- to during the influenza season
Gianino et al., 2017 [38]	Italy	2010–2013	Hospital database analysis (N = 5291–5544 across years studied)	All-cause absenteeism during influenza epidemic and non-epidemic periods	The average duration of absenteeism during the epidemic period increased among all employees by +2.07 days/person (range 2.99–5.06)
Zaffina et al., 2019 [39]	Italy	2016–2018	Retrospective observational study of healthcare workers (N = 2090–2097 across time periods studied)	All-cause absenteeism during influenza epidemic and non-epidemic periods	Absenteeism rate was 0.95–0.96 days greater in epidemic versus non-epidemic periods
Gianino et al., 2021 [40]	Italy	2010–2013 2017–2018	Healthcare worker absenteeism data from hospital database	Excess absenteeism in severe influenza season compared with non-epidemic period and with three moderate influenza seasons	Average number of days lost per week was 1263 in the severe epidemic and 898 in the non-epidemic period Absenteeism increased by +2.63 days during the severe epidemic versus non-epidemic period (relative increase 70%, from 4.05 to 6.68 days/person, *p* < 0.01) Excess absenteeism was greater during the severe versus moderate influenza seasons (+0.75 days/person, *p* = 0.03)
Huiberts et al., 2022 [41]	The Netherlands	2016–2019	Data on registered schoolteacher sick leave, vaccine effectiveness and incidence of ILI in primary care	Total absenteeism and self-reported influenza sick leave extrapolated to total teacher population based on incidence of ILI	Modeling predicted 46,479–62,966 influenza absenteeism notifications among the total teacher population of the Netherlands per year (23–31% of the total teacher population)

Abbreviations: ARI, acute respiratory infection; CDC, US Centers for Disease Control and Prevention; CI, confidence interval; ECDC, European Centre for Disease Prevention and Control; GP, general practitioner; ICPC, International Classification of Primary Care; ILI, influenza-like illness; PCR, polymerase chain reaction; RSV, respiratory syncytial virus; SD, standard deviation; SFOPH, Swiss Federal Office of Public Health.

**Table 2:** Studies on productivity loss due to influenza/ILI.

Publication	Location	Time Period	Study Design	Definition of Illness	Results
**General adult populations: laboratory-confirmed influenza**					
Petrie et al., 2016 [9]	United States	2012–2013	Prospective study of working adults (N = 1548)	Laboratory-confirmed influenza versus influenza negative	Influenza cases subjectively assessed their work productivity as impeded to a greater degree than influenza-negative cases (6.0 versus 5.4; *p* < 0.001; rating scale 1–10) 95% of influenza cases reported some degree of work productivity loss (score ≥1)
Van Wormer et al., 2017 [10]	United States	2012–2016	Analysis of data from participants in vaccine effectiveness studies (N = 1278)	PCR-confirmed influenza-positive versus confirmed influenza-negative people with ARI	Influenza was associated with significantly greater workplace productivity loss (rating scale 1–10) relative to non-influenza ARI (β ± SE = 11.1 ± 1.6, *p* < 0.001, univariate model)
Carrat et al., 2002 [14]	France	2000	Household contact study	Laboratory-confirmed influenza	Among 199 index cases with influenza, 9% did not stop working The median time until return to usual work activity for those who stopped working was 7 days (95% CI, 7–9)
**General adult populations: ILI**					
Carrat et al., 2004 [21]	France	1999–2000 influenza season	Observational household contact study (N = 701)	ILI	Median time taken to return to normal work activity was 7 days (range 6–7) if antibiotics taken and 7 (range 7–8) if antibiotics not taken
Palmer et al., 2010 [24]	United States	2007–2008 influenza season	Observational cohort study of employees (N = 2013)	ILI (modified CDC definition)	Employees with ILI were less productive for 2.5 h each day with ILI symptoms compared with 1.1 h for those with other wintertime illnesses Employees with ILI had significantly more hours of presenteeism than employees with other wintertime illnesses (1.4 [SD 3.7] versus 0.5 [SD 1.8], *p* < 0.001) Household members with ILI had significantly more hours of presenteeism than household members with other wintertime illnesses (1.3 [SD 3.5] versus 0.4 [SD 1.4], *p* < 0.001)
**Specific populations: ILI**					
De Perio et al., 2014 [25]	United States	2012–2013	Survey of school employees (N = 412)	ILI, defined as being sick with fever and either sore throat or cough	Of employees with ILI symptoms, 92 (77%) reported working while ill. Eight reported working <1 day, 60 reported working 1–3 days, and 22 reported working ≥4 days Presence of other medical conditions was not significantly associated with working while ill
Chiu et al., 2017 [33]	United States	2014–2015	Survey of healthcare workers (N = 1914)	ILI, defined as fever, sore throat or cough	Of 414 workers who reported ILI, 183 (41.4%) worked during their illness, for a median duration of 3 days (range 0–30)
Cowman et al., 2019 [7]	United States	2017–2018	Staff survey at Medical Center (N = 220)	ILI, defined as CDC definition of fever and ≥1 of cough, sore throat, nasal congestion or body aches	54% (n = 107) of house staff and 26% (n = 6) of program leaders reported presenting to work with ILI in the past 12 months
Jiang et al., 2019 [34]	Canada	2010–2014	Active surveillance of healthcare workers (N = 2093)	ARI (shortness of breath, cough, sore or scratchy throat, or coryza)	Among 1036 participants with ARI symptoms, 539 (52.0%) worked on every scheduled day and 980 (94.6%) worked ≥1 day The mean days worked with ARI was 1.93 (95% CI, 1.91–1.95)
Hoang Johnson et al., 2021 [35]	United States	2017–2018	Healthcare worker survey (N = 2391)	ILI, defined as symptoms of fever, chills, cough or sore throat	Of workers with ILI, 43% did not stay at home and 36% did not wait until being afebrile for at least 24 h without fever-reducing medication before returning to work

Abbreviations: ARI, acute respiratory infection; CDC, US Centers for Disease Control and Prevention; CI, confidence interval; ILI, influenza-like illness; PCR, polymerase chain reaction; SD, standard deviation; SE, standard error.

**Table 4:** Studies on cost of work loss due to influenza/ILI.

Publication	Location	Time Period	Study Design	Definition of Illness	Results
**General adult populations: physician-diagnosed influenza**					
Karve et al., 2013 [16]	United States	2005–2009	Retrospective analysis of employee data from MarketScan databases	≥1 medical claim with diagnosis of influenza	Total costs associated with influenza-related workplace absence per case of influenza ranged from USD 279.5 (2005–2006) to USD 226.3 (2006–2007)
Karve et al., 2013 [15]	United States	2000–2009	Retrospective analysis of employee data from MarketScan databases	Influenza-related medical encounters with temporal co-occurrence of workplace absence	The average per-patient cost associated with influenza-related workplace absence was USD 209.66 Costs were greater during influenza B vaccine-mismatched seasons than during matched seasons (mean: USD 237.31 versus USD 175.10) The cost of average influenza-related productivity losses per 100,000 plan members was USD 42,581 Higher costs of productivity losses were observed during mismatched seasons (USD 51,483 per 100,000 members) than during matched seasons (USD 31,454)
**General adult populations: ILI**					
Nettleman et al., 2001 [18]	United States	1998	Survey of parents of schoolchildren (N = 954)	Respiratory illness, excluding asthma	Of adults who missed work to care for an ill child, 167 (34%) sometimes or always lost wages and 329 (66%) used paid leave
Akazawa et al., 2003 [20]	United States	January–July 1996	Household survey (N = 7037)	ILI	Mean absenteeism costs because of ILI estimated at USD 137 per person in 1996 dollars
Li et al., 2007 [23]	United States	1996–2002	Medical Expenditure Panel Survey (N = 12,850 households)	ILI	In households with ILI, workdays lost as a result of members own illness cost an additional USD 143.36 compared with non-ILI households Cost of workdays lost caring for other family members was an additional USD 113.92 with insurance/full employment and USD 424.83 without insurance/employment.
Tomonaga et al., 2021 [27]	Switzerland	2016–2017	Absenteeism data from Swiss Sentinel Surveillance Network of the Swiss Federal Office of Public Health (SFOPH)	ILI, defined as sudden onset of high fever >38 °C and cough or sore throat, as well as secondary illnesses after influenza	Total costs of lost productivity due to inability to work were CHF 115 million in 2016 and CHF 103 million in 2017
Ferro et al., 2020 [44]	Italy	2017–2018	Observational cohort study of employees of a manufacturing company (N = 408)	Comparison of absenteeism during influenza epidemic and non-epidemic periods	Monthly mean cost of absenteeism per employee was higher for unvaccinated versus vaccinated individuals (EUR 129 versus EUR 54, *p* = 0.028)
Bridges et al., 2000 [43]	United States	1997–1999	Double-blind, placebo-controlled trial of inactivated influenza vaccine (N = 1184)	ILI, defined as feverishness or temperature of 37.7 °C plus cough or sore throat (CDC definition)	In 1997/1998, the cost of workdays lost due to ILI per person was USD 68.28 in the vaccine group and USD 47.05 in the placebo group In 1998/1999, the cost of workdays lost due to ILI per person was USD 19.40 in the vaccine group and USD 28.43 in the placebo group
**Healthcare workers: ILI**					
Szucs et al., 2001 [36]	Switzerland	1999–2000	Healthcare worker survey (N = 200)	ILI	Cost of productivity loss for the hospital estimated at CHF 1.2 million (range 0.68–2.0) for ILI and CHF 289,000 (range 164,000–480,000) for influenza infections per season, representing 0.3% and 0.08% of the annual expenditure of the hospital
**Healthcare workers: influenza surveillance trends**					
Zaffina et al., 2019 [39]	Italy	2016–2018	Retrospective observational study of healthcare workers (N = 2090–2097 across time periods studied)	Comparison of absenteeism during influenza epidemic and non-epidemic periods	The total cost of excess absenteeism in non-vaccinated healthcare workers was EUR 117,175.58 in 2016/2017 and EUR 134,884.76 in 2017/2018, based on an average daily cost of EUR 169.80
Gianino et al., 2019 [53]	Italy	2010–2013	Hospital data on absenteeism (N = 5401)	Sporadic absences (any cause) during influenza epidemic periods	Total cost of workdays lost/year in influenza epidemic periods was EUR 1,763,683

Abbreviations: CDC, Centers for Disease Control and Prevention; ILI, influenza-like illness; SFOPH, Swiss Federal Office of Public Health.

## Data Availability

Data included in this review article are those provided in published papers.

## References

[1] Keech M., Beardsworth P. (2008). The impact of influenza on working days lost: A review of the literature. Pharmacoeconomics.

[2] European Centre for Disease Prevention and Control (2022). Factsheet About Seasonal Influenza.

[3] Centers for Disease Control and Prevention (2023). Disease Burden of Flu.

[4] World Health Organization (WHO) Influenza (Seasonal). https://www.who.int/news-room/fact-sheets/detail/influenza-(seasonal).

[5] Krammer F., Smith G.J.D., Fouchier R.A.M., Peiris M., Kedzierska K., Doherty P.C., Palese P., Shaw M.L., Treanor J., Webster R.G. (2018). Influenza. Nat. Rev. Dis. Primers.

[6] Edwards C.H., Tomba G.S., de Blasio B.F. (2016). Influenza in workplaces: Transmission, workers’ adherence to sick leave advice and European sick leave recommendations. Eur. J. Public Health.

[7] Cowman K., Mittal J., Weston G., Harris E., Shapiro L., Schlair S., Park S., Nori P. (2019). Understanding drivers of influenza-like illness presenteeism within training programs: A survey of trainees and their program directors. Am. J. Infect. Control.

[8] Blanchet Zumofen M.H., Frimpter J., Hansen S.A. (2023). Impact of Influenza and Influenza-Like Illness on Work Productivity Outcomes: A Systematic Literature Review. Pharmacoeconomics.

[9] Petrie J.G., Cheng C., Malosh R.E., VanWormer J.J., Flannery B., Zimmerman R.K., Gaglani M., Jackson M.L., King J.P., Nowalk M.P. (2016). Illness Severity and Work Productivity Loss Among Working Adults With Medically Attended Acute Respiratory Illnesses: US Influenza Vaccine Effectiveness Network 2012–2013. Clin. Infect. Dis..

[10] Van Wormer J.J., King J.P., Gajewski A., McLean H.Q., Belongia E.A. (2017). Influenza and Workplace Productivity Loss in Working Adults. J. Occup. Environ. Med..

[11] Fragaszy E.B., Warren-Gash C., White P.J., Zambon M., Edmunds W.J., Nguyen-Van-Tam J.S., Hayward A.C., Flu Watch Group (2018). Effects of seasonal and pandemic influenza on health-related quality of life, work and school absence in England: Results from the Flu Watch cohort study. Influenza Other Respir. Viruses.

[12] Esposito S., Gasparini R., Bosis S., Marchisio P., Tagliabue C., Tosi S., Bianchi C., Crovari P., Principi N. (2005). Clinical and socio-economic impact of influenza and respiratory syncytial virus infection on healthy children and their households. Clin. Microbiol. Infect..

[13] Principi N., Esposito S., Marchisio P., Gasparini R., Crovari P. (2003). Socioeconomic impact of influenza on healthy children and their families. Pediatr. Infect. Dis. J..

[14] Carrat F., Sahler C., Rogez S., Leruez-Ville M., Freymuth F., Le Gales C., Bungener M., Housset B., Nicolas M., Rouzioux C. (2002). Influenza burden of illness: Estimates from a national prospective survey of household contacts in France. Arch. Intern. Med..

[15] Karve S., Meier G., Davis K.L., Misurski D.A., Wang C.C. (2013). Influenza-related health care utilization and productivity losses during seasons with and without a match between the seasonal and vaccine virus B lineage. Vaccine.

[16] Karve S., Misurski D.A., Meier G., Davis K.L. (2013). Employer-incurred health care costs and productivity losses associated with influenza. Hum. Vaccin. Immunother..

[17] De Blasio B.F., Xue Y., Iversen B., Gran J.M. (2012). Estimating influenza-related sick leave in Norway: Was work absenteeism higher during the 2009 A(H1N1) pandemic compared to seasonal epidemics?. Eurosurveillance.

[18] Nettleman M.D., White T., Lavoie S., Chafin C. (2001). School absenteeism, parental work loss, and acceptance of childhood influenza vaccination. Am. J. Med. Sci..

[19] Neuzil K.M., Hohlbein C., Zhu Y. (2002). Illness among schoolchildren during influenza season: Effect on school absenteeism, parental absenteeism from work, and secondary illness in families. Arch. Pediatr. Adolesc. Med..

[20] Akazawa M., Sindelar J.L., Paltiel A.D. (2003). Economic costs of influenza-related work absenteeism. Value Health.

[21] Carrat F., Schwarzinger M., Housset B., Valleron A.J. (2004). Antibiotic treatment for influenza does not affect resolution of illness, secondary visits or lost workdays. Eur. J. Epidemiol..

[22] Nichol K.L., D’Heilly S., Ehlinger E. (2005). Colds and influenza-like illnesses in university students: Impact on health, academic and work performance, and health care use. Clin. Infect. Dis..

[23] Li S., Leader S. (2007). Economic burden and absenteeism from influenza-like illness in healthy households with children (5–17 years) in the US. Respir. Med..

[24] Palmer L.A., Rousculp M.D., Johnston S.S., Mahadevia P.J., Nichol K.L. (2010). Effect of influenza-like illness and other wintertime respiratory illnesses on worker productivity: The child and household influenza-illness and employee function (CHIEF) study. Vaccine.

[25] De Perio M.A., Wiegand D.M., Brueck S.E. (2014). Influenza-like illness and presenteeism among school employees. Am. J. Infect. Control.

[26] Thorrington D., Balasegaram S., Cleary P., Hay C., Eames K. (2017). Social and Economic Impacts of School Influenza Outbreaks in England: Survey of Caregivers. J. Sch. Health.

[27] Tomonaga Y., Zens K.D., Lang P., Born R., Schwenkglenks M., Swiss Sentinel Surveillance Network (2021). Productivity losses due to influenza and influenza-like illness in Switzerland: Results of the Swiss Sentinel Surveillance Network in a non-pandemic era. Swiss Med. Wkly..

[28] Millot J.L., Aymard M., Bardol A. (2002). Reduced efficiency of influenza vaccine in prevention of influenza-like illness in working adults: A 7 month prospective survey in EDF Gaz de France employees, in Rhone-Alpes, 1996–1997. Occup. Med..

[29] Sessa A., Costa B., Bamfi F., Bettoncelli G., D’Ambrosio G. (2001). The incidence, natural history and associated outcomes of influenza-like illness and clinical influenza in Italy. Fam. Pract..

[30] Schanzer D.L., Zheng H., Gilmore J. (2011). Statistical estimates of absenteeism attributable to seasonal and pandemic influenza from the Canadian Labour Force Survey. BMC Infect. Dis..

[31] Groenewold M.R., Burrer S.L., Ahmed F., Uzicanin A., Luckhaupt S.E. (2019). Health-Related Workplace Absenteeism Among Full-Time Workers—United States, 2017-2018 Influenza Season. MMWR. Morb. Mortal. Wkly. Rep..

[32] Sartor C., Zandotti C., Romain F., Jacomo V., Simon S., Atlan-Gepner C., Sambuc R., Vialettes B., Drancourt M. (2002). Disruption of services in an internal medicine unit due to a nosocomial influenza outbreak. Infect. Control Hosp. Epidemiol..

[33] Chiu S., Black C.L., Yue X., Greby S.M., Laney A.S., Campbell A.P., de Perio M.A. (2017). Working with influenza-like illness: Presenteeism among US health care personnel during the 2014-2015 influenza season. Am. J. Infect. Control.

[34] Jiang L., McGeer A., McNeil S., Katz K., Loeb M., Muller M.P., Simor A., Powis J., Kohler P., Di Bella J.M. (2019). Which healthcare workers work with acute respiratory illness? Evidence from Canadian acute-care hospitals during 4 influenza seasons: 2010–2011 to 2013–2014. Infect. Control Hosp. Epidemiol..

[35] Hoang Johnson D., Osman F., Bean J., Stevens L., Shirley D., Keating J.A., Johnson S., Safdar N. (2021). Barriers and facilitators to influenza-like illness absenteeism among healthcare workers in a tertiary-care healthcare system, 2017-2018 influenza season. Infect. Control Hosp. Epidemiol..

[36] Szucs T.D., Ruef C., Muller D., Sokolovic E., Beeler I., Ostermayer W. (2001). The economic impact of influenza in a university hospital setting. Infect. Control Hosp. Epidemiol..

[37] Van Buynder P.G., Konrad S., Kersteins F., Preston E., Brown P.D., Keen D., Murray N.J. (2015). Healthcare worker influenza immunization vaccinate or mask policy: Strategies for cost effective implementation and subsequent reductions in staff absenteeism due to illness. Vaccine.

[38] Gianino M.M., Politano G., Scarmozzino A., Charrier L., Testa M., Giacomelli S., Benso A., Zotti C.M. (2017). Estimation of sickness absenteeism among Italian healthcare workers during seasonal influenza epidemics. PLoS ONE.

[39] Zaffina S., Gilardi F., Rizzo C., Sannino S., Brugaletta R., Santoro A., Castelli Gattinara G., Ciofi Degli Atti M.L., Raponi M., Vinci M.R. (2019). Seasonal influenza vaccination and absenteeism in health-care workers in two subsequent influenza seasons (2016/17 and 2017/18) in an Italian pediatric hospital. Expert Rev. Vaccines.

[40] Gianino M.M., Kakaa O., Politano G., Scarmozzino A., Benso A., Zotti C.M. (2021). Severe and moderate seasonal influenza epidemics among Italian healthcare workers: A comparison of the excess of absenteeism. Influenza Other Respir. Viruses.

[41] Huiberts A., van Cleef B., Tjon A.T.A., Dijkstra F., Schreuder I., Fanoy E., van Gageldonk A., van der Hoek W., van Asten L. (2022). Influenza vaccination of school teachers: A scoping review and an impact estimation. PLoS ONE.

[42] Nichol K.L., D’Heilly S., Ehlinger E.P. (2008). Influenza vaccination among college and university students: Impact on influenzalike illness, health care use, and impaired school performance. Arch. Pediatr. Adolesc. Med..

[43] Bridges C.B., Thompson W.W., Meltzer M.I., Reeve G.R., Talamonti W.J., Cox N.J., Lilac H.A., Hall H., Klimov A., Fukuda K. (2000). Effectiveness and cost-benefit of influenza vaccination of healthy working adults: A randomized controlled trial. JAMA.

[44] Ferro A., Bordin P., Benacchio L., Fornasiero F., Bressan V., Tralli V., Moretti F., Majori S. (2020). Influenza vaccination and absenteeism among healthy working adults: A cost-benefit analysis. Ann. Ig..

[45] King J.C., Cummings G.E., Stoddard J., Readmond B.X., Magder L.S., Stong M., Hoffmaster M., Rubin J., Tsai T., Ruff E. (2005). A pilot study of the effectiveness of a school-based influenza vaccination program. Pediatrics.

[46] King J.C., Stoddard J.J., Gaglani M.J., Moore K.A., Magder L., McClure E., Rubin J.D., Englund J.A., Neuzil K. (2006). Effectiveness of school-based influenza vaccination. N. Engl. J. Med..

[47] Bleser W.K., Miranda P.Y., Salmon D.A. (2019). Child Influenza Vaccination and Adult Work Loss: Reduced Sick Leave Use Only in Adults With Paid Sick Leave. Am. J. Prev. Med..

[48] Speroni K.G., Dawson E., Atherton M., Corriher J. (2005). Influenza vaccination: Incidence of symptoms and resulting absenteeism in hospital employees. AAOHN J..

[49] Antinolfi F., Battistella C., Brunelli L., Malacarne F., Bucci F.G., Celotto D., Cocconi R., Brusaferro S. (2020). Absences from work among healthcare workers: Are they related to influenza shot adherence?. BMC Health Serv. Res..

[50] Murti M., Otterstatter M., Orth A., Balshaw R., Halani K., Brown P.D., Hejazi S., Thompson D., Allison S., Bharmal A. (2019). Measuring the impact of influenza vaccination on healthcare worker absenteeism in the context of a province-wide mandatory vaccinate-or-mask policy. Vaccine.

[51] Costantino C., Casuccio A., Caracci F., Bono S., Calamusa G., Ventura G., Maida C.M., Vitale F., Restivo V. (2019). Impact of Communicative and Informative Strategies on Influenza Vaccination Adherence and Absenteeism from Work of Health Care Professionals Working at the University Hospital of Palermo, Italy: A Quasi-Experimental Field Trial on Twelve Influenza Seasons. Vaccines.

[52] Waite N.M., Pereira J.A., Houle S.K.D., Gilca V., Andrew M.K. (2022). The impact of influenza on the ability to work, volunteer and provide care: Results from an online survey of Canadian adults 50 years and older. BMC Public Health.

[53] Gianino M.M., Politano G., Scarmozzino A., Stillo M., Amprino V., Di Carlo S., Benso A., Zotti C.M. (2019). Cost of Sickness Absenteeism during Seasonal Influenza Outbreaks of Medium Intensity among Health Care Workers. Int. J. Environ. Res. Public Health.

[54] Puig-Asensio M., Douglas M., Holley S., Kukla M.E., Abosi O., Mascardo L., Carmody B., Gent C., Diekema D.J., Hartley P. (2019). Impact of expanded influenza post-exposure prophylaxis on healthcare worker absenteeism at a tertiary care center during the 2017-2018 season. Infect. Control Hosp. Epidemiol..

[55] Centers for Disease Control and Prevention (2023). People at Higher Risk of Flu Complications.

